# Influence of Random Plasmonic Metasurfaces on Fluorescence Enhancement

**DOI:** 10.3390/ma15041429

**Published:** 2022-02-15

**Authors:** Veronica Anăstăsoaie, Roxana Tomescu, Cristian Kusko, Iuliana Mihalache, Adrian Dinescu, Catalin Parvulescu, Gabriel Craciun, Stefan Caramizoiu, Dana Cristea

**Affiliations:** National Institute for Research and Development in Microtechnologies—IMT Bucharest, 126A, Erou Iancu Nicolae Street, 077190 Voluntari, Romania; veronica.anastasoaie@imt.ro (V.A.); cristian.kusko@imt.ro (C.K.); iuliana.mihalache@imt.ro (I.M.); adrian.dinescu@imt.ro (A.D.); catalin.parvulescu@imt.ro (C.P.); gabriel.craciun@imt.ro (G.C.); stefan.caramizoiu@imt.ro (S.C.); dana.cristea@imt.ro (D.C.)

**Keywords:** fluorescence, fluorescence enhancement, plasmonic metasurface, nanophononics, localized field enhancement

## Abstract

One of the strategies employed to increase the sensitivity of the fluorescence-based biosensors is to deposit chromophores on plasmonic metasurfaces which are periodic arrays of resonating nano-antennas that allow the control of the electromagnetic field leading to fluorescence enhancement. While artificially engineered metasurfaces realized by micro/nano-fabrication techniques lead to a precise tailoring of the excitation field and resonant cavity properties, the technological overhead, small areas, and high manufacturing cost renders them unsuitable for mass production. A method to circumvent these challenges is to use random distribution of metallic nanoparticles sustaining plasmonic resonances, which present the properties required to significantly enhance the fluorescence. We investigate metasurfaces composed of random aggregates of metal nanoparticles deposited on a silicon and glass substrates. The finite difference time domain simulations of the interaction of the incident electromagnetic wave with the structures reveals a significant enhancement of the excitation field, which is due to the resonant plasmonic modes sustained by the nanoparticles aggregates. We experimentally investigated the role of these structures in the fluorescent behaviour of Rhodamine 6G dispersed in polymethylmethacrylate finding an enhancement that is 423-fold. This suggests that nanoparticle aggregates have the potential to constitute a suitable platform for low-cost, mass-produced fluorescent biosensors.

## 1. Introduction

There is currently a growing need in the biomedical field to find reliable, fast and low-cost methods for monitoring intermolecular interactions, cancer detection and cell study. Methods such as ELISA, mass spectrometry or polymerization chain reaction (PCR) techniques are expensive due to high reagent consumption and the need for qualified personnel [1]. An alternative to these approaches is to use fluorescence (FL) biosensors because many of the traditional bioimaging and biodetection methods are based on fluorescence. A method to increase the response of the biosensors is to employ the effect of metasurfaces upon the fluorescence enhancement (FLEN) offering the advantages of a fast and sensitive detection for various biological samples such as proteins and antibodies [2,3,4,5,6,7,8,9,10,11], DNA [12,13,14,15,16,17], cells [18,19], and cancer biomarkers [20,21,22,23]. In his way, biosensors based on metasurfaces can be developed and optimized according to the desired application.

Essentially, metasurfaces are nano-platforms consisting of periodic arrays of metallic or dielectric nano-antennas, of various shapes and sizes sustaining resonances [24], patterned on a substrate, on which fluorophores are deposited. Metasurfaces have been already used for the fabrication of flat and lightweight optical components, such as gratings, lenses, beam shapers (holographic phase masks, colour filters, and absorbers) [25,26,27,28,29,30], but also for biosensors [31,32,33,34,35], solar cells, solar shields and photodetectors [36,37,38,39,40,41,42,43,44,45,46,47,48,49,50,51,52,53]. In the last few years, it was demonstrated that they have the potential to control both the propagation and the emission of light at the nanoscale as well.

Therefore, due to their salient properties, metasurfaces were intensively investigated as platforms for FLEN. Specifically fluorescent and light-emitting metasurfaces can be obtained by covering them with layers containing quantum dots (QDs), dye molecules, and by integrating direct bandgap semiconductors into the metasurface architecture [54,55,56]. Moreover, FLEN with metasurfaces for two cyanine dyes (Cy3 and Cy5) can be obtained by depending on the structure morphology [57]. More recently, the employment of dielectric metasurfaces was studied to improve the resolution of a fluorescence microscope [58]. The role played by the metasurfaces in FLEN is twofold: firstly, due to the plasmonic resonance, the excitation electromagnetic field is enhanced in the proximity of the nanoantennas; secondly, due to the Purcell [59] effect appearing in the electromagnetic cavities of the metasurfaces, the radiative recombination rate is increased contributing further to the FLEN [60].

A method to enhance the FL is to pattern metallic nanostructures on a substrate obtaining a structure appropriate for enhancing the intensity of localized electromagnetic fields, and thus increase the excitation characteristics of the emitting fluorophores placed in their proximity. Although this approach is straightforward, it presents the disadvantage that it is time consuming and uses expensive technological processes to obtain a large area platform with nanostructures with the desired geometry. Furthermore, there is a strong dependence of the FLEN with respect to the nanostructure sizes and shapes. For instance, FLEN occurs in the case of spherical geometries, whereas for nanorods-like patterns, a significant quenching effect appears [61]. An opportunity to circumvent this problem is to employ in-house metasurface structures, a novel concept in nanophotonics which have the role of tailoring the shape of the optical fields by nanostructuring the substrates [62]. This type of structure is a subclass of the metamaterials and can be considered to include advanced effective materials in the sense that the sizes of the nano-antennas (meta-atoms) are much smaller than the wavelength of the field, such that they can be considered homogeneous.

To date, fluorescence emission intensification analysis has been performed most frequently using one of the following methods: (i) metallic nanostructures (gold, silver or aluminium) patterned on a substrate; and (ii) NP deposited on a functionalized surface. Using these methods, distance between the nanostructure and the fluorophore and also the degree of NP aggregation can be controlled to achieve the desired outcome [63].

A tailored metasurface can increase up to 450-fold the FL intensity (of the dye molecules in NIR) in comparison with a flat surface [56]. This paper presents low-cost metasurfaces consisting of aggregates of random distributions of metallic nanoparticles of gold, aluminium and silver on silicon and glass substrates, which offer a fluorescence enhancement factors (up to 423) comparable with results achieved by artificially engineered structures. We present the technological processes needed to obtain these configurations, which consists of deposition of ultrathin metallic films (discontinuous or annealed to obtain nanostructures) on silicon and glass substrates. The obtained nanoparticle aggregates were characterized from the morphological point of view in order to find out the geometric parameters (shapes, sizes and configurations). We performed a thorough experimental investigation of the FLEN dependence upon the geometrical parameters of the metasurfaces, as well as on the materials employed for their fabrications with the final aim to obtain large areas (1.5/2 cm^2^) and low-cost metasurfaces composed of nanostructures that have resonance modes corresponding to the fluorescence absorption and emission spectra of the chromophore Rhodamine 6G dye. This work was not a microscopical analysis, but rather a phenomenological one with the aim of achieving plasmonic resonances and field enhancement at a wavelength that overlaps the excitation wavelength of the fluorophore. The purpose of our experimental investigations was to demonstrate that fluorescent enhancement can be obtained on large areas using a low-cost process to obtain cheap platforms for sensing applications for biosensing applications in the visible spectral domain.

## 2. Materials and Methods

The substrates used in this study are optical glass and silicon wafers (100, SIEGERT WAFER GmbH, Aachen, Germany). The sample sizes were 2 × 2 cm^2^ for the glass substrate and 1.5 × 1.5 cm^2^ for the silicon substrate. The substrate cleaning protocol involved several steps with an application time of 10 min each: (a) ultrasound in Extran solution (Extran^®^ MA 02 Neutral, Merck KGaA, Darmstadt, Germany), (b) rinsing with deionized water, (c) treatment in piranha solution (H_2_SO_4_:H_2_O_2_ = 3:1), (d) rinsing with deionized water, and e) drying in isopropyl alcohol. After cleaning, the samples were covered with metal thin films. All metals were deposited by e-beam evaporation on our deposition equipment: TEMESCAL FC-2000 (Temescal, Livermore, CA, USA). In all cases, the base pressure was 5 × 10^−7^ Torr and the deposition rate 0.1 Å/s. To obtain nanostructured metal layer acting as a plasmonic metasurface we deposited both discontinued layers (very thin- 2–4 nm-thick mass equivalent) or continuous layers that were nanostructured by thermal annealing.

The thermal annealing to define random metasurface nanostructures from thicker continues metallic layers was performed using a programmable hot place HP61 (from Torrey Pines Scientific) at a temperature of 250 °C for 30/60 min, at a controlled room temperature of 22 °C and 40% humidity. These treatments were applied to silver layers with 15, 20 and 30 nm thickness to obtain meta-atom structures.

Films of Rhodamine 6G dye (R6G, 252433, Sigma-Aldrich, St. Louis, MO, USA) dispersed in polymethylmethacrylate (MICROCHEM 950 PMMA A4 from Kayaku Advanced Materials, Inc., Westborough, MA, USA) were coated on top of the nanostructured metallic films. We tested three concentrations of C_1_ = 50 µM, C_2_ = 10 µM and C_3_ = 5 µM dye in in solution of PMMA 4% in anisole. The PMMA-R6G layer was deposited by spin coating using SUSS MicroTec LabSpin 8 (SUSS MicroTec SE, Garching bei München, Germany) employing three deposition speeds (3000, 4500 and 6000 rpm).

The polymer thickness was measured by employing the ellipsometry method using SE 800 XUV spectroscopic ellipsometer (Table 1).

The morphological characterizations of the samples were carried out using scanning electron microscopy (SEM- Field Emission un Scanning Electron Microscope (FEG-SEM) -Nova NanoSEM 630 (FEI Company, Hillsboro, OR, USA) and Electron Beam Lithography and nanoengineering workstation—e_Line-Raith GmbH, Dortmund, Germany) and atomic force microscopy (AFM—Scanning Near-field Optical Microscope—Witec alpha 300S, Witec, Ulm, Germany).

Steady-state photoluminescence emissions (and optical absorption measurements) were carried out using a fluorescence spectrometer FLS920 (Edin. Inst. Ltd., Livingston, UK) equipped with a Xe 450 W arc lamp as excitation source. The emission of the samples was measured for an excitation of λ = 480 nm. The spectra show an emission band centred around 550 nm, which was characteristic of the rhodamine 6G thin films (see Section 3).

For the modelling and simulation of plasmonic metasurfaces employed in FLEN, we utilized the Finite-difference time-domain (FDTD) method with the aid of the OptiFDTD commercial software (OptiWave, Ottawa, ON, Canada). More details about the simulation parameters employed when tailoring a metasurface structure for FLEN can be seen in [64].

The investigated materials and parameters are presented in Table 2.

**Figure 1:**
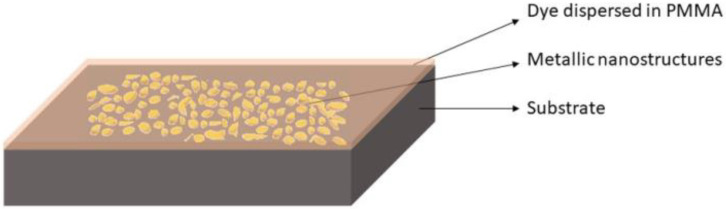
Fluorescence enhancement platform based on a random plasmonic metasurface.

Figure 1 presents the artist view of the proposed platforms based on random plasmonic metasurface, designed for FLEN.

## 3. Results and Discussions

To understand the fluorescence enhancement in our samples, we examined the structures formed by the deposition of the thin metallic films. Essentially, they can be regarded as aggregates of coupled metallic nanoparticles, presenting random distribution of sizes, shapes and configurations, which, in some cases, show clear spatial separations, while in other cases they exhibit short range order percolations. Since these nanoparticles present resonant plasmonic modes (as it is seen in the simulations), the fluorescence enhancement [65] can be described in terms of two contributing phenomena: the local electromagnetic field enhancement occurring between nanoparticles leading to an increase of the excitation rate and the increase of radiative decay due to the Purcell effects. Another phenomenon present in these structures, which is detrimental to fluorescence, is the energy transfer from the fluorescent molecules to the metallic nanoparticle leading to non-radiative decay. This phenomenon is dependent on the distance between the chromophore and the metallic particles, such that for a close proximity between them the fluorescence is suppressed leading to quenching. Therefore, the fluorescence response of the nanoparticle aggregates and chromophore configurations results from the interplay between the aforementioned phenomena, such that for achieving the FLEN it is necessary to optimize the structures in terms of both geometrical and material parameters of the structures.

To obtain low-cost FLEN platforms, we started our study by investigating the influence of an ultra-thin discontinuous metallic films on the fluorescence emission. The metals selected for this analysis are gold (Au), aluminium (Al), and silver (Ag).

### 3.1. Modelling and Simulation of Plasmonic Metasurfaces Employed in FLEN

**Figure 2:**
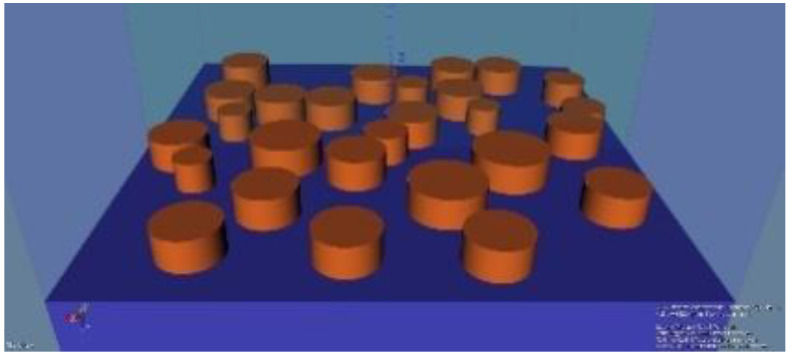
The supercell with a random distribution of nanopillars.

We performed simulations to evaluate the enhancement of the excitation field and of the fluorescence emission as well, due to plasmonic resonances appearing between the meta-atoms of the random metasurface. To determine whether the proposed structures offer localized electromagnetic field enhancement and plasmonic resonances, which can increase the excitation rate of fluorophores on a silicon substrate, we numerically analyzed a metallic metasurface using a three-dimensional (3D) finite-difference time-domain (FDTD) method. The simulation of a random structure is not a trivial task. To overcome this challenge, we designed a supercell (Figure 2), which consisted of a random distribution of nanocylinders with random diameters, imposing periodic boundary conditions with the mention that this can induce an artificial periodic effect. We consider that this effect does not significantly modify the results regarding the field enhancement due to the large number of nanopillars contained in the supercell. On the propagation direction (*Z*), the absorbing perfectly matched layer (PML) conditions were employed to minimize the reflections that could occur when the incident radiation touches the boundary.

**Figure 3:**
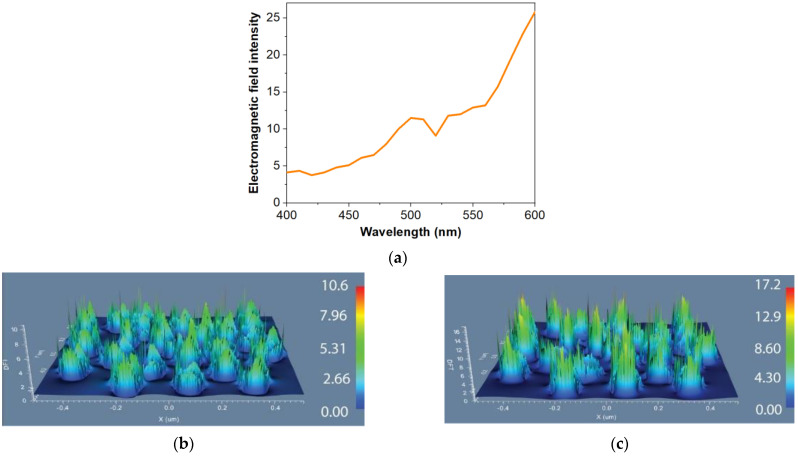
(**a**) Electromagnetic fields average enhancement as a function of wavelength: (**b**) Field configurations for a silver random metasurface at the wavelength of 480 nm; (**c**) Field configurations for a silver random metasurface at the wavelength of 550 nm.

Considering the experimental results (see Section 3.2) we selected to numerically investigate the electromagnetic field enhancement obtained with a random metasurface with silver meta-atoms. The height of the simulated meta-atoms is 60 nm the average height of silver nanostructures measured from SEM characterizations, and the selected diameters are between 70 nm and 150 nm. The FDTD simulations were performed using continuous wave (CW) at specific wavelengths from the absorption and emission spectra of Rhodamine 6G. Employing the average field enhancement between the nanopillars resulted from each simulation we obtained a full spectrum of the electromagnetic field enhancements to demonstrate the origin of the different fluorescence enhancements (Figure 3a). Moreover, Figure 3b,c shows the localized electromagnetic field offered by the metasurfaces. We observed that at 480 nm wavelength (the excitation of R6G employed in our experiments), the electromagnetic field presents a seven fold average enhancement with a maximum of 10 (see Figure 3b). At 550 nm wavelength (the emission of R6G), the field shows an average enhancement of thirteen (Figure 3a) with a maximum enhancement of seventeen (see Figure 3c). We note that the enhancement was considered by averaging over the fields present in the spaces between nanoparticles composing the supercell.

The simulation results demonstrate that these type of structures presents resonant plasmonic modes leading to local electromagnetic field enhancement at both the excitation and emission of R6G. This effect will lead to an increase of the excitation rate when the chromophores are in the proximity of the metasurface.

### 3.2. Experimental Results

We started the experiments by investigating the fluorescent response of a bare sili-con substrate coated with a R6G:PMMA, which was selected as a reference (for each con-centration) in order to identify the enhancement factor offered by the metasurface structures. The enhancement factor is calculated as the ratio between the maximum enhancement value and the reference intensity (see Table 3).

**Figure 4:**
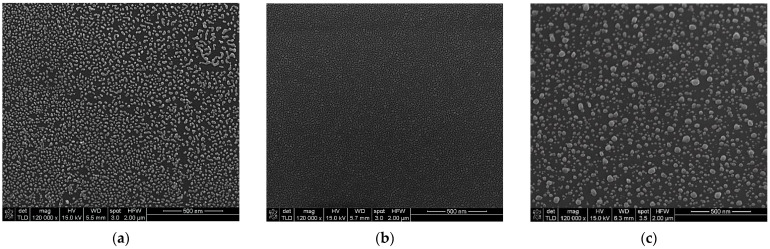
SEM micrographs of a 2 nm metallic (mass equivalent): (**a**) Au, (**b**) Al, (**c**) Ag.

The first random metasurfaces analyzed consisted of a 2 nm-thick (mass equivalent) metallic discontinuous film deposited on silicon substrate, as shown in Figure 4 SEM micrographs. The Au layers show clustered structures with smaller diameters, unevenly dispersed. The SEM micrographs show that Al presents random structures with a monodisperse pattern, while the Ag layer has nanoparticles of different sizes placed at various distances.

**Figure 5:**
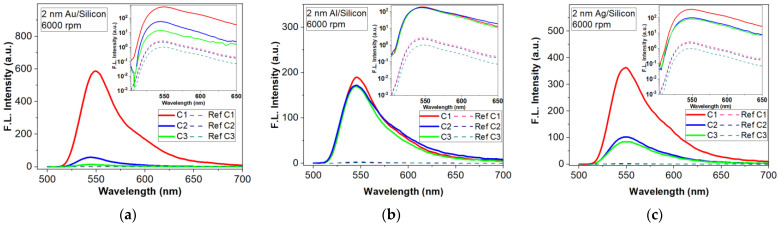
Florescence intensity enhancement by a 2 nm metallic (mass equivalent) films on silicon substrate (inset are the FLEN with logarithmic y scale): (**a**) Au meta-atoms; (**b**) Al meta-atoms; (**c**) Ag meta-atoms.

On top of the metallic nanostructures, layers of PMMA doped with R6G in three different concentrations were deposited. Figure 5 shows the fluorescence intensity of R6G in the presence of metallic metasurfaces in comparison with the reference. In the inset of the figures, a graph in the logarithmical scale is presented in order to show the magnitude orders. The improvement of fluorescence by Au metasurfaces is small for lower concentrations of fluorophores in the PMMA layer (Figure 5a). Only for high concentrations of R6G in PMMA (C_1_) was a significant improvement of fluorescence emission observed. The same behaviour can be seen in the case of Ag metasurfaces (Figure 5c). In the case of aluminium metasurfaces, the concentration has a very low influence on the improvement of the fluorescence emission (Figure 5b). This is due to the really thin nanostructures (hights between 3 and 5 nm, see Table 3) that can accommodate, nearby, only a limited number of fluorophore molecules, approximatively independent of the dye concentration. The advantage of these Al structures is that they offer the best improvement of fluorescence intensity for the thinnest layers of fluorophore dispersed in PMMA.

**Figure 6:**
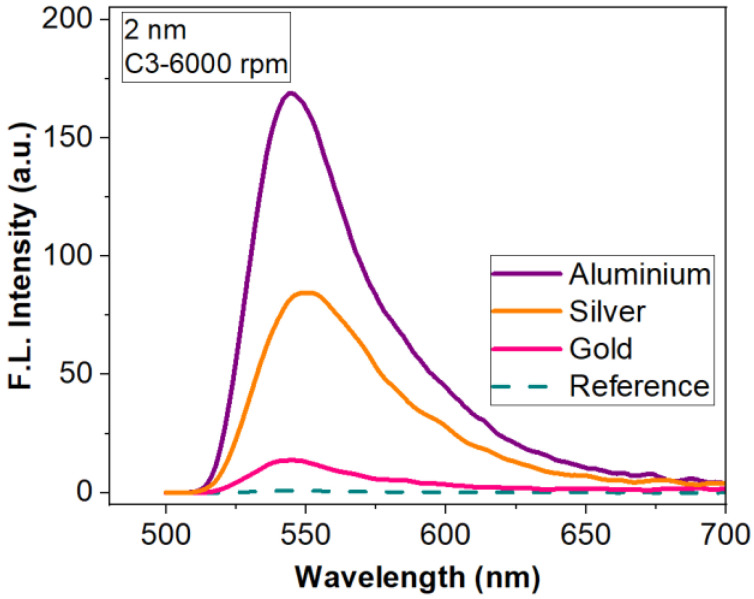
Fluorescence enhancement for three types of metasurface (nanostructured 2 nm-thick Al, Ag and Au thin films) for R6G:PMMA- C_3_ thin film deposited at 6000 rpm.

Figure 6 shows a comparison of the fluorescence emission obtained with the three types of metal layers and bare silicon as a reference. The compared results were obtained for R6G:PMMA layers of the same thickness (deposited with an 6000 rpm rate for 40 s) and concentration (C_3_). These layers were selected to demonstrate that we can achieve important fluorescence enhancement for low dye concentrations in thin films. It can be seen that the metasurface with Al elements offers the best response in fluorescence compared to other metals. FLEN is influenced by the distance between the aluminium nanoparticles, as well as their heights and diameters, which are almost constant on the sample surface, offering the possibility for most plasmonic resonances to properly interact and increase the fluorescent intensity. Moreover, the improvement of the fluorescence emission by the presence of the metasurface is considerable for all types of metal.

The highest enhancement factor (of 204, Figure 6) is obtained with the metasurface, which presents monodispersion, clear spatial separation and almost periodically distribution of the nanostructures (Al meta-atoms) on the silicon substrate after deposition (Figure 4b). In this case, the distance between the metallic nanoparticles, as well as their heights and diameters, offer the possibility for most plasmonic resonances to properly interact, resulting in a multitude of localized field enhancements points evenly distributed on the surface. Meanwhile, the higher distances observed in the cases of Au and Ag films result in a smaller intensity, offering lower enhancement factors (17.3 and 102).

**Figure 7:**
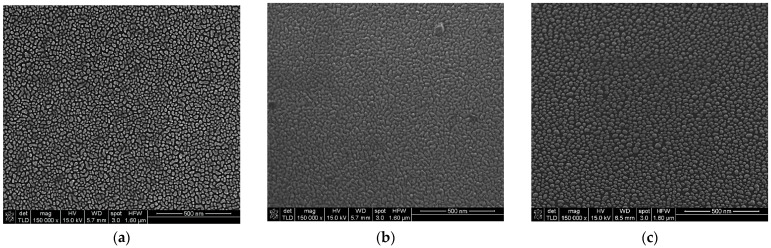
SEM micrographs of a 4 nm metallic (mass equivalent): (**a**)—Au, (**b**)—Al, (**c**)—Ag.

The next investigation was on a metasurfaces composed of meta-atoms obtained by depositing a 4 nm-thick (mass equivalent) metallic film. SEM micrographs for this deposited thickness also show different morphologies specific to each metal (Figure 7). It can be seen that in the case of Au and Al, the meta-atoms present short range order percolations. The Ag metasurface has well-defined elements of different dimensions uniformly arranged on the silicon surface, a pattern similar with 2 nm mass-equivalent Al layer.

**Figure 8:**
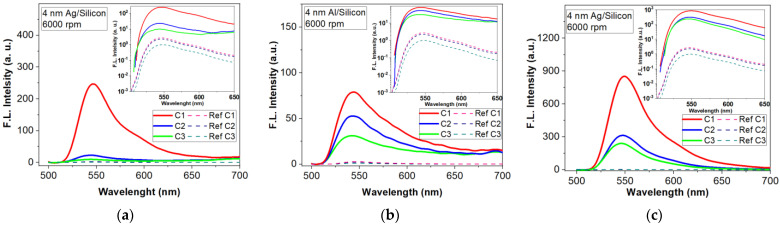
Florescence intensity enhancement by a 4 nm metallic (mass equivalent) films on silicon substrate (inset are the FLEN with logarithmic y scale): (**a**) Au meta-atoms; (**b**) Al meta-atoms; (**c**) Ag meta-atoms.

The influence of the metallic meta-atoms on fluorescence intensity for the three concentrations is presented in Figure 8. For all three metals the improvement depends on the concentrations of the R6G dispersed in PMMA. For Au metasurfaces, fluorescence intensity is small for low concentrations (C_2_, C_3_) as one can see in Figure 8a. Only for the highest concentration C_1_ a significant fluorescence emission enhancement can be noticed. Figure 8b,c indicate that Al and Ag-based metasurfaces improve the fluorescence for all three concentrations of R6G in PMMA; however, the Ag-based metasurface shows an enhancement factor with almost an order of magnitude higher than in the case of Al.

**Figure 9:**
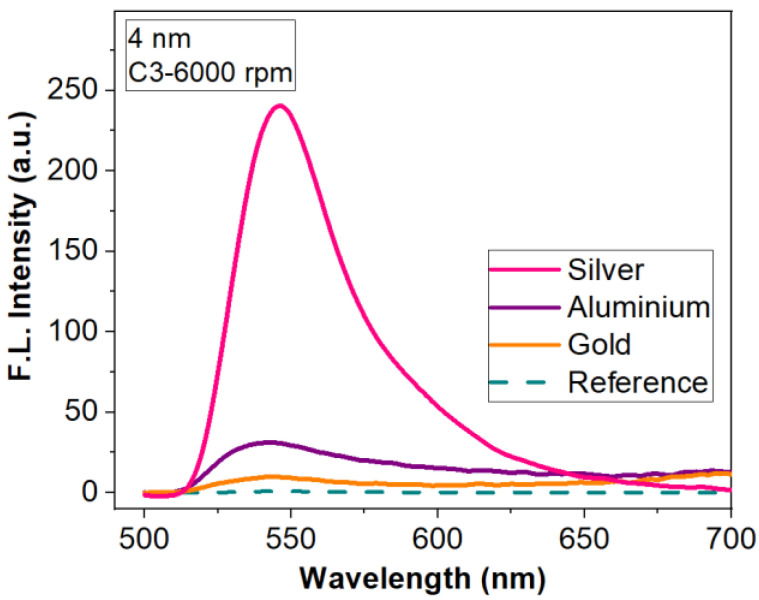
Fluorescence enhancement for tree types of metasurfaces (nanostructured 4 nm-thick Al, Ag and Au thin films) for R6G:PMMA- C_3_ thin film deposited at 6000 rpm.

Thus, florescent emission intensity is higher (289 fold) when using an Ag metasurface due to the geometry and the spatial separation of the meta-atoms. For Au and Al, the presence of percolations increases the radiative decay, the effect being a quenching of the fluorescence intensity, showing small improvements of 12- and 38-fold, respectively. Figure 9 presents the influence of the metasurface structures on the fluorescence emission spectrum for C_3_—R6G:PMMA deposited with a rate of 6000 rpm for 40 s.

The results show a clear dependence of the fluorescence enhancement on geometry and distribution of the metallic nanostructures on the silicon surface.

**Figure 10:**
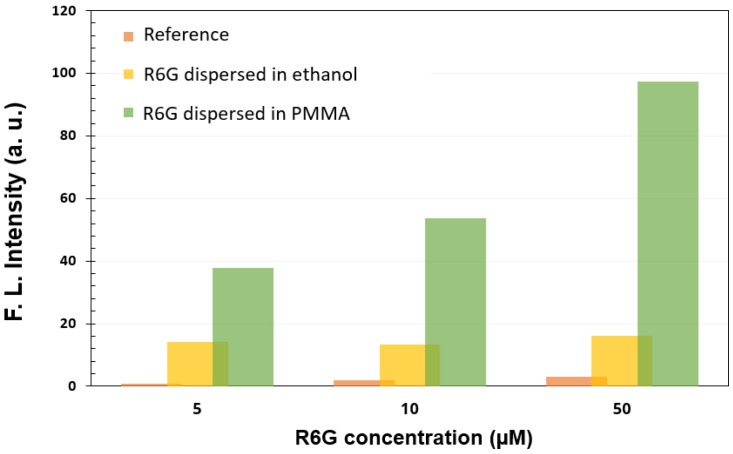
PMMA influence on FLEN.

To highlight the advantage of fluorophore dispersion in PMMA, we also analyzed the intensity of fluorescence emission for R6G dispersed in ethanol and deposited on 4 nm-thick Al–based metasurfaces. In Figure 10, one can that even if the fluorescent response of the R6G/ethanol layer coated on a metallic metasurface is higher than if we have only the silicon substrate, the intensity obtained when R6G was dispersed in PMMA is much higher. The role of PMMA is to protect the fluorophore luminescent properties from degradation when drying in normal atmosphere.

**Figure 11:**
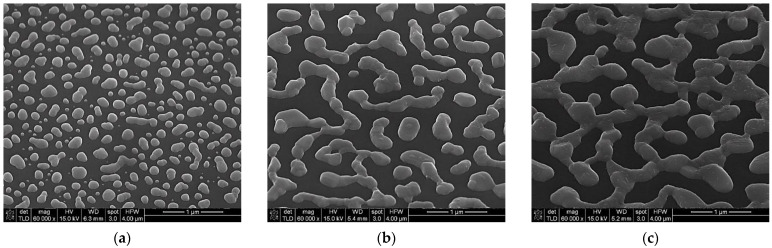
SEM micrographs of thick Ag layers after the specific thermal annealing performed to obtain the metallic meta-atoms: (**a**)—15 nm, (**b**)—20 nm, (**c**)—30 nm.

Considering the experimental results and simulations for metasurfaces based on 60 nm-high Ag nanostructures, we continued the investigation using layers with higher thickness (15, 20, 30 nm). In order to obtain individual meta-atoms, the deposited layers were subjected to a thermal annealing, after which the silver was aggregated forming nanostructures of different geometries and sizes. All treatments were performed at a temperature of 250 °C, for 30 min (15 nm) or 60 min (20 and 30 nm). After the annealing, the initial layer of 15 nm presents individual flattened hemispheres with diameters between 40 and 260 nm and heights between 50 and 90 nm (Table 3). The 20 nm annealed layer presents short order percolated nanostructures with dimensions between 200 and 600 nm and heights between 80 and 110 nm (Table 3). The morphology of the 30 nm layer obtained after the annealing has a like-mesh appearance with an average height of about 120 nm (Table 3). Figure 11 presents the SEM micrographs of these three silver layers after annealing.

**Figure 12:**
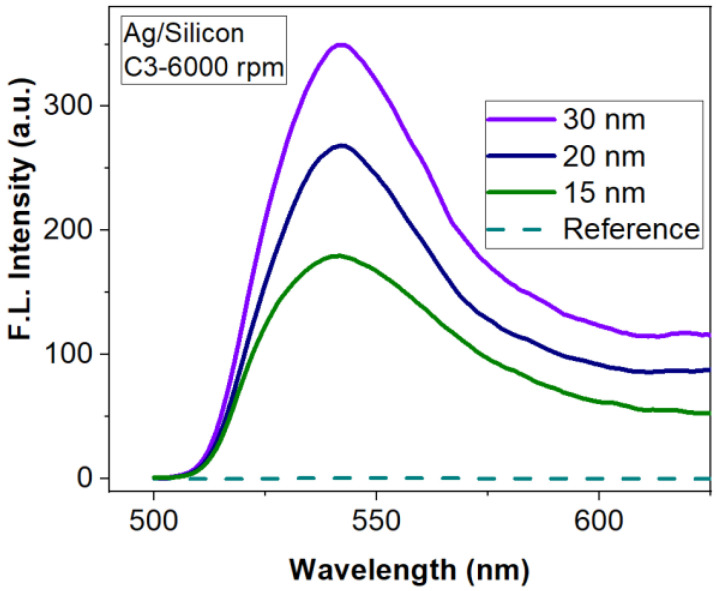
Influence of Ag metasurfaces/silicon substrate on FLEN.

Figure 12 shows the influence of different Ag meta—atoms patterned on a silicon substrate on fluorescence emission intensity. The enhancement factor increases with the height of the silver nanostructures because the number of interacting dye molecules in the proximity of the metallic nanostructures increases as well. In this case, the 30 nm annealed layer offers the best intensity enhancement (Table 3) because the heights of the nanopillars are comparable with thickness of the R6G:PMMA layer, so almost all the fluorophore molecules can interact with the plasmonic resonances.

**Figure 13:**
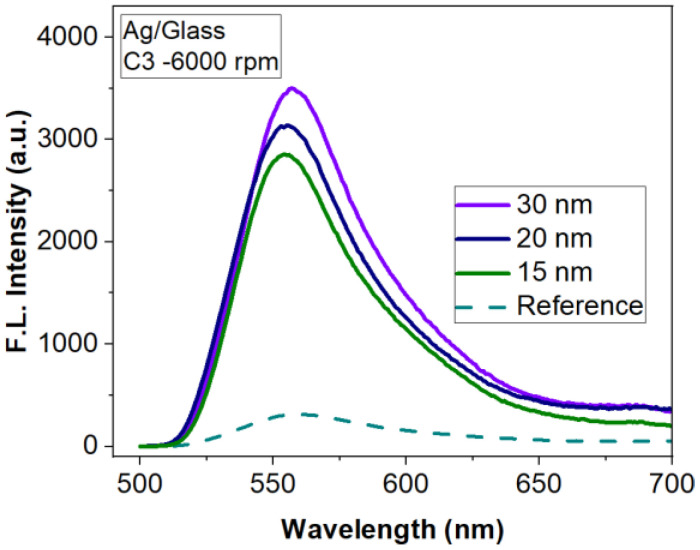
Influence of Ag metasurfaces/glass substrate on FLEN.

Taking into account the results obtained on silicon substrate, we continued the study of the FLEN on glass substrate for the metallic layers with thicknesses of 15, 20 and 30 nm Ag prepared in the same conditions as before. The results are presented in Figure 13. As one can see, the fluorescence intensity improvement factor reaches values up to 11 times for the 30 nm layer compared to the reference (R6G:PMMA on glass).

The obtained results are summarized in Table 3.

The results for metasurfaces obtained by depositing and annealing a layer of Ag show that silicon substrate brings a better improvement in fluorescence than the glass substrate for all concentrations investigated. However, even for glass substrates, the fluorescence enhancement obtained with Ag-based metasurfaces is significant and useful in biosensing applications.

Another important factor in obtaining an optimal improvement for the fluorescence intensity is the distance between the fluorophore particles and the metallic structures [63,66]. Thus, due to the high oxidation rate of silver when it comes into contact with the atmosphere, an oxide layer is formed on its surface, which allows the separation of fluorophore from the metal components. This distance leads to the minimization of the quenching effect and to a good energy transfer between the fluorescent molecules and the nanostructures. The reverse effect is observed in the case of Al, where the distance is smaller and therefore FLEN has lower values, but also in the case of Au, where the fluorophore is in direct contact with the metal, resulting in very low values of FLEN due to the suppression of the fluorescence.

## 4. Conclusions

To demonstrate the improvement of fluorescence emission by metasurface structures, the spatial and geometric parameters of randomly arranged meta-atoms were numerically analyzed. The influence of metallic nanostructures placed on silicon/glass substrates on the fluorescence intensity was studied in comparison with flat substrates. For this study, we selected the metals most frequently employed in the development of plasmonic structures (gold, aluminium, silver). We used three concentration of Rhodamine 6G dispersed in PMMA coated on top of metasurface structures.

Analysing the structures obtained by depositing discontinuous ultrathin metallic films, we found that an Al metasurfaces obtained from a 2 nm-thick mass equivalent layer offer a 204 fold enhancement of the fluorescence emission, while Au- and Ag-based metasurfaces provide an enhancement factor of 17.3 and 102, respectively. For the 4 nm-thick (mass equivalent) metallic films, the best FLEN is attained with Ag (an improvement of 289 fold), while Au and Al show relatively low values (12 and 38 fold fluorescence enhancement). These values were obtained for C_3_ concentration and 6000 rpm for 40 s deposition rate of the R6G:PMMA layer.

By investigating the role of thicker Ag layers (15, 20, 30 nm—thin films nanostructured by thermal annealing) on fluorescence, we demonstrated that the enhancement factor increases with the height of the nanostructures. The size of the structures compensates the radiative decay, which can appear due to the morphology obtained after annealing.

The study is proof that the electromagnetic field enhancement is dependent on the shape and distance between the metasurface elements. Moreover, the distance between the fluorophore particles and the metallic structures plays an important role in obtaining an optimal improvement for the fluorescence intensity. This fact was highlighted by the values for FLEN factor obtained for Ag, which presents a high oxidation rate when in contact with atmosphere, leading to the formation of an oxide layer before the deposition of the R6G:PMMA layer.

The results demonstrate that we can achieve an intensification of the fluorescence emission for R6G fluorophore on two of the most frequently used substrates in biodetections (silicon and glass) depending on the geometry and distribution of the metallic nanostructures. The purpose of our experimental investigations was to demonstrate that fluorescent enhancement can be obtained on large areas using a low-cost process to obtain cheap platforms for sensing applications in the visible spectral domain.

## Figures and Tables

**Table 1:** R6G:PMMA layer thickness.

Deposition Rate (rpm)	Thickness (nm)
3000	233 ± 4
4500	187 ± 3
6000	169 ± 5.5

**Table 2:** Materials and parameters.

Substrate	Investigated Metals	Metallic Film Mass Equivalent Thickness	R6G Concentration	Deposition Speed (rpm)
Glass and Silicon	Aluminium	2/4 nm	C_1_ = 50 µM	-
	Gold		C_2_ = 10 µM	3000 and 6000
	Silver	2/4/15/20/30 nm	C_3_ = 5 µM	-

**Table 3:** Fluorescence enhancement factor attain on metallic films deposited on different substrates.

Substrate	Metal	Layer Thickness/Mass Equivalent (nm)	Real Thickness of the Nanostructures (nm)	F. L. Intensity (a.u.)		Enhancement Factor
				Reference with C3	Metasurface with C3	
Silicon	Au	2	7–12	0.8302	14.33	17.3
		4	11–16	0.8302	9.96	12
	Al	2	3–5	0.8302	169.18	204
		4	10–12	0.8302	31.17	38
	Ag	2	8–13	0.8302	84.49	102
		4	12–17	0.8302	240	289
		15	50–90	0.8302	180	217
		20	80–110	0.8302	269.51	325
		30	106–140	0.8302	350.77	423
Glass	Ag	15	-	314.1964	2850.74	9
		20	-	314.1964	3137.23	10
		30	-	314.1964	3499	11

## Data Availability

The data presented in this study are available on request from the corresponding author.
